# Labourers' Wages and Labourers' Homes

**Published:** 1888-05-26

**Authors:** Earl Grey

**Affiliations:** K.G., G.C.M.G.


### LABOURERS' WAGES AND LABOURERS' HOMES.

By the Right Honourable Earl Grey, K.G., G.C.M.G.

*(Concluded from p. 106.)*

Towards the close of the year 1883 there was much discussion in the newspapers and elsewhere on the general wretchedness of the dwellings of the poor in the East of London, and on the steps that ought to be taken to secure for them better habitations. Various plans for effecting such an improvement were proposed, and something at least has, I believe, been done with advantage for providing improved lodging for some of the working classes in that part of the town; but though it is now more than four years since attention was strongly called to the subject, it does not appear that the number of those who are still inhabiting houses of the worst class has been seriously diminished, or that their general mode of living shows a greater regard than formerly for the comforts and decencies of civilised life. More decided measures than any that have yet been taken are therefore clearly wanted, and I retain the opinion I expressed in two letters to the *Times*, in November, 1883, that the policy most likely to succeed in bringing about an improvement in the present state of things would be that of imposing a pecuniary responsibility upon the lessors if they allowed houses or rooms let by them by the week or month to be overcrowded or occupied without a due regard to decency and to sanitary arrangements. This responsibility might be imposed by making the lessors of these dwellings liable to a very moderate fine (say 5*s.*), to be summarily levied upon the lessor for every week that a room could be shown to have been improperly occupied. This would make it the interest of the lessor to prevent the violation by his tenants of whatever regulations might be established as to the occupation of their dwellings, and he would have sufficient power to do so since the dwellings of the lower classes of working men in London are almost universally held by them as weekly tenants, who could therefore be removed if they refused to conform to the rules laid down for them. In imposing such a responsibility upon the lessors of dwellings it would be necessary to arm them with more prompt and effectual means of protecting themselves against the damage of their property by their tenants than are afforded by the existing law, as there is no doubt that great and wanton damage is often done by tenants for which no sufficient remedy can be obtained; but with this precaution it would be just and reasonable to require every man who derives an income from providing dwellings for the poor to do so under such conditions as may be necessary for the public good.

In order to make a law for the purpose I have described really effectual, it would further be necessary to provide securities for its being duly enforced, but this might easily be done by means there is no occasion for now discussing. I am quite aware that the introduction of a stringent system of regulations with regard to the dwellings of the poor would be attended with very serious difficulty, but this difficulty would be diminished if the change were brought somewhat gradually instead of abruptly into operation. With this view it would be expedient that any law for making the lessors of dwellings taken by the week or month liable to fines for the violation of sanitary regulations by their tenants, should provide that this new liability should only take effect with regard to tenancies entered upon after the passing of the law, and should not apply in the case of persons still occupying rooms or houses in which they were dwelling when the law was passed. Such a proviso would be just in itself, and would tend to smooth the way for the introduction of the proposed reform, and yet it would not very greatly retard its coming into full operation from the frequency with which the class of the population concerned in it are known to change their abodes.

In November, 1883, I proposed the change in the law I have just described, in the belief that it would abate, though it could not cure, the evils arising from the over-crowding of a large number of the London population in dwellings unfit for civilised habitation. I still believe that it would answer this purpose to a considerable extent, and that it would likewise tend gradually to diminish the over-competition for work in the eastern part of the town. If those from whom the poorest class of workmen hire their dwellings were made liable to a weekly fine for every room which they allowed to be overcrowded or improperly occupied, they would not be able to receive new tenants without requiring from them higher rents than are now usually paid. It is only by crowding greater numbers into their houses than they ought to contain, and by refusing to expend upon them the money necessary to keep them in decent repair, that those who make a business of letting the worst kind of lodgings are able to make it pay them at the rents they obtain. If a well-enforced law compelled them to give accommodation of a less miserable character to their tenants, the better accommodation they would have to give must necessarily be paid for (at least in his first instance) by those who obtained it.

But it is the possibility of finding shelter in such miserable dens as many of those which are now inhabited which is one of the main causes of the too great influx of population into London, if therefore an effective restraint were imposed on their being received in such abodes some of those who now keep crowding into an already overstocked market for labour would be forced to seek elsewhere for a livelihood; emigration and looking for work where more can be found would be their natural resources. With a growing trade, a check thus given to the coming of additional labourers to London would naturally lead to a gradual rise of wages. The daily rate of pay of labourers might not be increased, but as the existing disproportion between the amount of employment that is available and the number of those who seek it became less, the annual earnings of labourers would be increased by their being less often thrown out of work than they now are. The cost of living in such a manner as to satisfy what are regarded by labourers as their indispensable wants determines the minimum rate of wages, because if less can be earned the supply of labour falls off. If, therefore, labourers were not allowed to occupy lodgings in which the requirements of health and decency are disregarded, but were called upon to pay for dwellings of a better description, their other necessary expenses remaining the same as at present, the minimum of wages for unskilled labour must be increased. Imposing a strict pecuniary responsibility for the proper conditions of dwellings on those from whom they are hired would thus tend indirectly, but effectually, to bring about a raising of the standard of living and of the wages received by the lowest class of London workmen.

The whole cost of providing better dwellings for this class might not, however, require to be met by charging them with higher rents; the effectual prevention of overcrowding would probably prevent the competition for houses in certain situations, and for land to build them upon, from being as intense as at present, and the owners of the property would in consequence find it necessary to reduce the ground rents they receive from those who hold houses under them to be sub-let to the actual occupiers of the working classes. This would be no small addition to the advantages to be anticipated from the proposed measure, since its effect would be to prevent an excessive income from being drawn from land in special situations at the cost of much evil to the public. By imposing, as I have suggested, pecuniary responsibility for the proper condition of houses and rooms on those from whom they are immediately held by the occupiers, and by also making more effectual provision than now exists for preventing new houses from being built of faulty construction and arrangements, much I believe would be done towards preventing hereafter that overcrowding of London which is to be regarded as the main cause of the difficulties and distress to which many of its inhabitants are now exposed.

In order to give full effect to the policy I have suggested some further measures would be required, and to which it is unnecessary now to advert, but I may observe that among the objects to which these measures should be directed, I regard as the most pressing that of checking the influx of immigrants of the character of those who have for a considerable time been arriving here in large and increasing numbers. It has been proved that there are now coming to our shores, and chiefly to London, from the least civilised parts of Europe, great numbers of the lowest of their inhabitants, whose ignorance, habits of life, and want of means to support themselves, render their presence a source of grievous injury to our own people of the working class. A general and, as I think, most just demand has in consequence arisen for the adoption of some effectual means for protecting the country against this invasion, and various modes of doing so have been suggested. It has been proposed to prohibit the landing in England of emigrants from foreign countries who could not show that they had a suitable home to go to, and some adequate means of subsistence, the captains of vessels who landed passengers in violation of this prohibition being made subject to fines. Another proposal is that vessels bringing over emigrants from Europe in such large numbers that they might be presumed to be of a low class should be subject to a poll tax on the passengers they brought over, the proceeds of this tax being made over to the parishes chiefly burdened by this immigration. A good deal might be said in favour of each of these proposed measures, and especially of the first, which appears to hold out a prospect of considerable advantages, but on the other hand there are some obvious difficulties which would be likely to arise if either were adopted. Whether these difficulties would not admit of being guarded against, and whether some different and better mode of dealing with the subject might not be suggested, are questions on which I will not venture to express an opinion, but I can have no doubt that if the necessity of offering a barrier to the invasion of this country by the lowest of the population of other nations is recognised (as I think it must be), some means of accomplishing that object will be found here, as they have been in the United States, where an example has been set which ought not to be lost upon us. The inquiry now in progress by a Committee of the House of Lords will, it is to be hoped, succeed in pointing out what changes in the law would be most likely to answer the purpose in view.

